# Tiotropium Bromide in Chronic Obstructive Pulmonary Disease and Bronchial Asthma

**DOI:** 10.14740/jocmr2305w

**Published:** 2015-09-25

**Authors:** Alcibey Alvarado-Gonzalez, Isabel Arce

**Affiliations:** a Internal Medicine and Neumology, Clinica de Diagnostico Medico, San Jose, Costa Rica; b Medicine and General Surgery, Clinica de Diagnostico Medico, San Jose, Costa Rica

**Keywords:** Tiotropium bromide, Chronic obstructive pulmonary disease, Bronchial asthma

## Abstract

Inhaled bronchodilators are the mainstay of pharmacological treatment for stable chronic obstructive pulmonary disease (COPD), including β_2_-agonists and muscarinic antagonists. Tiotropium bromide, a long-acting antimuscarinic bronchodilator (LAMA), is a treatment choice for moderate-to-severe COPD; its efficacy and safety have been demonstrated in recent trials. Studies also point to a beneficial role of tiotropium in the treatment of difficult-to-control asthma and a potential function in the asthma-COPD overlap syndrome (ACOS). Combination of different bronchodilator molecules and addition of inhaled corticosteroids are viable therapeutic alternatives. A condensation of the latest trials and the rationale behind these therapies will be presented in this article.

## Introduction

In the 19th century, in the early 1800s, the British Army medical officers serving in India introduced anticholinergics in Western medicine [1]. The leaves and roots of *Datura stramonium* (source of stramonium), *Hyoscyamus niger* (source of hyoscine or scopolamine) and *Atropa belladonna* (which contains the alkaloid atropine), had all their place in most pharmacopoeias [2]. Given the liposolubility of these natural anticholinergics, they are easily absorbed across oral and respiratory mucous membranes. High-dose usage as bronchodilator generated side effects such as decreased salivation, dry mouth, mydriasis, blurred vision, urinary retention, decreased gut mobility, nausea, tachycardia and decreased sweating. The discovery of adrenergic agonists in the 1920s displaced these agents as the first-line treatment for asthma and emphysema [3].

Since the early 1970s, there has been a renewed interest in the usage of anticholinergic medication. This is due to several facts, such as the increase of prevalence, morbidity and mortality of asthma, the need to develop alternatives to therapy with β-agonists, a better understanding of the cholinergic mechanisms controlling airway caliber in states of health and disease and the development of synthetic analogues of atropine [4]. The new anticholinergic agents are water-soluble, poorly absorbed quaternary ammonium compounds, causing only mild systemic side effects when administered by inhalation. These agents include: ipratropium, thiazinamium, oxitropium, glycopyrronium, aclidinium and tiotropium bromide [5].

Worldwide, asthma and COPD affect the lives of approximately 300 and 200 million people, respectively [6]. Also, these are costly diseases to manage, for instance, the annual attributable cost of COPD in 2010 sum up to $36 billion in the US alone [7]. In spite of the current availability of evidence-based treatment guidelines, an effort needs to be made to continuously examine new and innovative approaches that will ensure the delivery of the best care possible to patients [8]. This paper summarizes novel evidence of tiotropium in chronic obstructive lung diseases.

## Molecular Biology

Muscarinic receptors have been classified into five subtypes, initially based on drug selectivity, and subsequently confirmed by molecular cloning. M_1_, M_2_ and M_3_ receptors are found in human airways [3]. M_1_ receptors are found in alveolar walls and in the parasympathetic airway ganglia, their blockade reduces the bronchoconstriction response. M_2_ receptors are located on postganglionic cholinergic nerve endings, and these auto-receptors limit the magnitude of vagally induced bronchoconstriction. M_3_ receptors are located on airway smooth muscle and submucosal glands, where they mediate bronchoconstriction and mucus secretion [4].

Muscarinic acetylcholine receptors are G protein-coupled receptors (GPCRs). M_1_ and M_3_ are coupled with G_q_ proteins, while M_2_ are coupled with G_i/o_ proteins [9]. Tiotropium bromide is an inhaled long-acting muscarinic antagonist (LAMA). It has a high affinity and dissociates very slowly from M_1_ and M_3_ receptors, and more rapidly from M_2_ receptors. It produces long-term blockade of cholinergic neural bronchoconstriction in human airways, providing 24-h bronchodilatation [10].

## Tiotropium in COPD Smokers

COPD is a major cause of morbidity and mortality, and many people suffer from this disease for years and die prematurely from it or its complications [8, 11]. Cigarette smoking is the most important single risk factor for COPD in the developed world. Treatment of COPD is now aimed at immediately relieving and reducing the impact of symptoms, as well as reducing the risk of future adverse health events, such as exacerbations [12]. In this section, the role of tiotropium in COPD smokers will be discussed.

The UPLIFT trial is a 4-year randomized placebo-controlled study, and it was performed in moderate to very severe COPD patients treated once a day with 18 µg inhaled tiotropium (dry powder, HandiHaler^®^). The study showed significant improvement in lung function and health-related quality of life and a reduction of exacerbations and hospitalization. But tiotropium did not significantly reduce the rate of decline in FEV_1_ or mortality compared to placebo (P ≤ 0.09) [13].

The results could be explained by several reasons. One possible explanation is that tiotropium does not influence the decline in lung function over time. In this context, data suggest that the symptomatic and functional improvement arise from mechanisms other than those identified to prolong life. It could also be that factors not yet been identified have an influence on mortality, and that they do not respond to tiotropium therapy [13].

It is also important to consider that, in the design of the UPLIFT study, the high rate of simultaneous prescription of other respiratory drugs may have affected the decline in lung function. This has been described as a ceiling effect, in which further improvements are not observed in the absence of an intervention that repairs or regenerates lung tissue [13]. The group of patients who received tiotropium but did not receive inhaled glucocorticoids (IGCs) or long-acting β_2_ agonists (LABAs), did show a statistically significant improvement in the decline rate in FEV_1_ (P ≤ 0.046), supporting this justification [14]. Another explanation is the higher rate of discontinuation in the placebo group. Patients who discontinued treatment had, on average, significantly more severe airflow obstruction at the beginning of the study. Consequently, those in the placebo group that completed the study may represent “healthy survivors” [13, 14].

In the UPLIFT study, tiotropium was associated with a decrease in respiratory morbidity (dyspnea and risk of respiratory failure) and cardiac morbidity (heart failure and acute myocardial infarction). Additionally, tiotropium was not associated with an increased incidence of pneumonia or stroke, opposite to what was previously reported in a meta-analysis [13, 15]. These findings were supported by the Food and Drug Administration (FDA) [16]. Nevertheless, there were still some concerns about its cardiovascular safety, and some researchers raised the need for controlled and randomized studies with greater statistical power [17].

The TIOSPIR trial is a randomized double-blind study involving 17,135 COPD patients. The study compared patients treated with Respimat^®^ in inhaler (daily doses of 2.5 and 5.0 µg) to a control group using HandiHaler^®^ (18 µg daily). In this trial, Respimat^®^ was not inferior to HandiHaler^®^ regarding risk of death, nor was it better than HandiHaler^®^ regarding the time of first exacerbation. In fact, comparative risk ratios were very close to 1, with 95% confidence intervals (CIs). In other words, the three doses with two different devices are comparable in terms of safety and efficacy [18].

In addition to the statistical power of the sample, one of the most relevant aspects of the TIOSPIR study is that it enrolled a substantial number of patients with cardiac conditions (1,825 with arrhythmia and 3,152 with ischemic heart disease, coronary artery disease or heart failure). The tiotropium Respimat^®^ did not increase the risk of death or adverse events in these patients. The tiotropium HandiHaler^®^ was associated with a reduced mortality, even in patients with coexisting heart disease [14, 19].

Former meta-analysis and observational studies identified up to a 52% increased risk of cardiovascular mortality in COPD patients treated with Respimat^®^, particularly in patients who had persistent arrhythmia, cardiomegaly or chronic renal failure [20]. Some authors even recommended that Respimat^®^ should not be prescribed to patients with COPD [21], but these authors subsequently endorsed TIOSPIR results [22]. Respimat^®^ generates a fine aerosol cloud (Soft Mist Inhaler) that moves very slowly (4 - 10 times slower than a pressurized inhaler), with a high proportion of the emitted dose deposited in the lung [23]. It was then presumed that a high systemic exposure could occur, which would explain the apparent increase in mortality; nonetheless, pharmacokinetic studies show similar systemic exposure to the drug, regardless of the delivery system [23, 24].

The results of TIOSPIR show that caution should be exercised in interpreting the safety outcomes of meta-analysis and observational studies; these are merely *post hoc* studies without a prior hypothesis [16]. Randomized clinical trials balance the confounding factors of observational studies, and lead to more realistic outcomes [21, 25].

## Tiotropium Mechanism of Action in COPD

COPD is an inflammatory disease, and during exacerbations a further intensified inflammatory response occurs. As mentioned, tiotropium has been shown to prolong the time to first exacerbation, compared to placebo, in addition to reducing the frequency of exacerbations and associated hospitalization [13, 18]. It is known that acetylcholine increases neutrophil chemotactic activity in COPD, and that this effect is attenuated *in vitro* by tiotropium, suggesting a possible anti-inflammatory mechanism, though it remains controversial [26].

A British study including 142 patients, randomized either to receive tiotropium or placebo for 1 year, failed to demonstrate a reduction in airway or systemic inflammatory markers. Of the three sputum inflammatory markers: interleukin-6 (IL-6), interleukin-8 (IL-8) and myeloperoxidase, none was found to be reduced after tiotropium therapy; in fact, IL-8 levels were found to be increased. This results could be attributed to the fact that tiotropium causes reduced production of mucus in the airway, increasing the concentration of cytokines. This also suggests that the measurement of cytokines in sputum is not an optimal method to assess airway inflammation [27].

Tiotropium reduces the volume of secretions, but does not alter the viscoelastic properties of mucus. *In vitro* studies show that acetylcholine induces the release of inflammatory mediators, such as the granulocyte-macrophage colony stimulating factor (GM-CSF), leukotriene B4 (LTB4) and prostaglandin E2 of epithelial cells. This release is mediated via muscarinic receptors that could be inhibited by tiotropium [28]. In the British study mentioned above, levels of LTB4 or GM-CSF were not measured, and they seem to be more relevant to evaluate cholinergic effects.

There is also evidence that tiotropium may prevent contractility and proliferation of airway smooth muscle cells and fibroblast proliferation. These findings support the hypothesis that the cholinergic system has a role in the pro-fibrotic processes of airway remodeling. Peribronchiolar fibrosis may be a key event in the progressive FEV_1_ decline in COPD [29]. Any inhibitory effect of tiotropium in the development of fibrosis may be detectable only after several years of treatment, as a slow decline in lung function [30]. Long-term studies are not available, but clinical evidence points towards muscarinic antagonists having an anti-inflammatory effect and/or an effect on airway remodeling in COPD.

## Tiotropium Compared to Other LAMA

For a decade, only the LAMA tiotropium bromide was available to clinicians and patients. That picture changed in 2012 with the approval of two new LAMAs: glycopyrronium bromide and aclidinium bromide. The three LAMAs are considered therapeutic options in the recent update of the global initiative for chronic obstructive lung disease (GOLD) [12].

Glycopyrronium bromide is a synthetic quaternary ammonium compound, which has been used for many years to reduce secretions and block vagal cardiac reflex [31]. Recently, a dry powder formula was developed, requiring once daily administration. After early promising preclinical and clinical results, a phase III trial called glycopyrronium bromide in COPD airways (GLOW) was conducted. These studies showed that inhaled glycopyrronium 50 µg once daily improves FEV_1_, dyspnea and health status [32]; a reduction in the number of exacerbations was also observed [33].

The GLOW2 study was developed to evaluate the efficacy and safety of glycopyrronium versus placebo and tiotropium. A faster onset of action of glycopyrronium compared to tiotropium was observed (5 min difference). There was also an improvement in the FEV_1_ AUC_0 - 4 h_ with glycopyrronium on day 1 and week 26; although these differences were statistically significant (57 mL, P ≤ 0.001 and 50 mL, P ≤ 0.01, respectively), the absolute values did not reach the minimal clinically important difference of 100 cc to impact clinical indexes [34]. The study was not designed to show statistical superiority over tiotropium, and usage of tiotropium was open label.

The GLOW3 trial showed that glycopyrronium is superior to placebo regarding exercise tolerance, with improvements in exercise endurance increasing over the 3 weeks study time [35]. Glycopyrronium has an acceptable safety profile, and it has a low incidence of cardiac and anticholinergic side effects. Consequently it has been considered as an alternative LAMA [32, 36].

Aclidinium bromide has been approved in the European Union (EU) and in the United States (US) as a maintenance treatment for COPD. Aclidinium needs to be administered twice daily, delivered via a multidose dry powder inhaler [36]. The BID regimen does not seem disadvantageous compared to once daily tiotropium and glycopyrronium. This regimen could be of particular benefit for patients with morning and evening symptoms, and it could also provide better nighttime bronchodilator relief [37, 38].

In phase III trials, aclidinium (compared to placebo) significantly improved lung function, dyspnea and health status in the first 24 weeks of treatment; later studies have shown that this was maintained for up to 52 weeks. The frequency of exacerbations was significantly reduced compared to placebo. Systemic bioavailability was low, with a reduced tendency to induce cardiac arrhythmias, indicating an adequate tolerability profile [36]. Further studies are required to assess the maximum clinical potential of this drug, but current results indicate a promising alternative.

## Tiotropium and LABA

The POET is a 1-year, randomized, double-blind, double-dummy and parallel-group trial, comparing the effect of treatment with 18 µg of tiotropium once daily versus 50 µg of salmeterol twice daily. The results indicated that in patients with moderate to very severe COPD, tiotropium is significantly more effective (P ≤ 0.001) than salmeterol in preventing exacerbations. Overall, the incidence of adverse events was similar with both drugs [39].

Indacaterol is the first LABA approved for once a day usage (ULTRA-LABA) in COPD. It was designed to have a quick onset of action (5 min), and ultra-long-acting duration, so it could be prescribed once daily. Indacaterol was initially approved in the EU in 2009 at doses of 150 - 300 µg; followed by 150 µg in Japan (2011), China (2012), and 75 µg in the US (2011) [40]. To date, indacaterol has been approved in more than 100 countries. This drug provided superior bronchodilator activity than the LABA formoterol (12 µg twice daily), with similar clinical outcomes. Indacaterol works at least similarly to tiotropium [12, 41].

## Dual Bronchodilator Therapy

Dual bronchodilator therapy is a recent strategy. β_2_-adrenoreceptors are coupled to a stimulatory G protein of adenylyl cyclase. This enzyme increases the intracellular cAMP concentration, leading to activation of protein kinase A (PKA). PKA activates the myosin-light-chain phosphatase, which in turn inactivates MLCK. MLCK cannot phosphorylate myosin, there is no interaction between actin and myosin, and subsequently smooth muscle contraction does not occur. Moreover, PKA phosphorylates the IP3 receptor, decreasing the release of calcium from the ER and limiting muscle contraction. On the other hand, muscarinic antagonists block the activation of MLCK by a different mechanism, and inhibit the synthesis of IP3 from PIP2 [10]. These two mechanisms explain at least partially their synergism.

It is also important to consider that sympathetic activity is predominant during daytime, whereas the parasympathetic system is more active at night; thus, control over both systems might provide beneficial effects. Another important consideration is the availability of devices that allow the simultaneous delivery of LABAs and LAMAs. This strategy facilitates adherence to treatment, enhances the bronchodilator effect and reduces side effects associated with high doses of a single drug. Dual bronchodilator therapy is superior to monotherapy, and it has been recommended by GOLD [12].

Adding a second bronchodilator to patients whose symptoms are under-controlled with monotherapy improves lung function, symptomatology and health status, without increasing risk of side effects. This was demonstrated in the INTRUST-1 and INTRUST-2 (indacaterol plus tiotropium versus tiotropium) and the GLOW6 (indacaterol plus glycopyrronium versus indacaterol) trials [42, 43]. Also, the administration of a fixed-dose combination (150 µg indacaterol plus 50 µg glycopyrronium) was demonstrated superior to single component administration [44]. The Ultibro^®^ Breezhaler^®^ inhalation device (indacaterol plus glycopyrronium) has been approved in the US, Japan and other countries in 2013.

The SPARK trial evaluated the effect of dual, long-acting inhaled bronchodilator treatment in severe and very severe COPD, randomizing patients to either a fixed-dose combination of indacaterol 110 µg plus glycopyrronium 50 µg, glycopyrronium 50 µg or tiotropium 18 µg (open label). The study showed that the dual bronchodilator is superior in preventing moderate to severe exacerbations compared with the single glycopyrronium and single tiotropium. These results indicate the potential of dual bronchodilator as a treatment for patients with severe and very severe COPD [45].

Ellipta^®^, a dry powder combination inhaler containing umeclidinium bromide (an LAMA) and vilanterol trifenatate (an LABA), was approved 13 months ago in the US and 7 months ago in the EU for maintenance treatment of COPD. This combination was shown to improve lung function, dyspnea, quality of life related to health and to reduce exacerbations, without adding serious adverse effects. Long-term investigations are needed to assess the effect of the drug on disease progression and to compare it directly to other fixed-dose combinations [46].

The European Commission has granted the marketing authorization for Striverdi^®^ (olodaterol), a long- and fast-acting bronchodilator, for the treatment of mild to severe COPD. TOviTO^®^, a large global phase III trial, is been conducted to investigate the efficacy and safety of the fixed-dose combination of tiotropium and olodaterol, delivered via the Respimat^®^ inhaler [18]. In May 2015, the FDA approved Stiolto^TM^ Respimat^®^ inhalation spray (olodaterol/tiotropium) as once daily maintenance treatment for COPD.

Long-term studies are needed to evaluate the role of LABA-LAMA combination therapy in disease progression, exacerbation, hospitalization and mortality. It is likely that patients in GOLD group B and those with reduced lung function (FEV_1_ of less than 50% predicted) and infrequent exacerbations (C1 and D1) are candidates for these combinations [46].

## LAMA, LABA and IGCs Combination Therapy

The ILLUMINATE study compared the fixed-dose combination of indacaterol/glycopyrronium (110/50 µg once daily) to fluticasone/salmeterol (500/50 µg twice daily) over 26 weeks in 523 patients with moderate-to-severe COPD without exacerbations in the previous year. The results showed a significant, sustained and clinically meaningful improvement in lung function with the once daily dual bronchodilator [47].

The WISDOM trial, a 12-month, double-blind, parallel group study, included 2,485 patients with severe COPD and a history of exacerbation. All patients received triple therapy consisting of tiotropium (at a dose of 18 µg once daily), salmeterol (50 µg twice daily) and fluticasone (500 µg twice daily) during a 6-week run-in period. Patients were then randomly assigned to continued triple therapy or withdrawal of fluticasone in three steps over a 12-week period. Glucocorticoid withdrawal met the non-inferiority criterion of 1.20 with respect to the first moderate to severe exacerbation, but in the upper limit of the 95% CI and with an HR of 1.06; 95% CI, 0.94 to 1.19. Also, there was a statistically significant decrease in FEV_1_ during the final step of glucocorticoid withdrawal, and there were minor changes in health status. There were no differences in the number of pneumonia cases [48].

The INSTEAD trial, a 26-week, double-blind, double-dummy, parallel-group, phase IV study, investigated the effect of switching 581 patients with moderate COPD and low risk of exacerbations from salmeterol/fluticasone to indacaterol monotherapy (current management guidance suggests these patients should not receive IGS). The results suggest that in this subgroup of patients, a once daily LABA produces similar changes in lung function to a twice daily LABA plus IGS. There were no statistically significant differences in the exacerbation rate, dyspnea index, use of rescue medication or number of serious adverse events [49, 50].

There have always been concerns about the balance of risks and benefits of using IGS in COPD patients [50]. Consistent evidence indicates that the long-term use of fluticasone-based IGS is associated to an increased risk of pneumonia [51, 52]. Similarly, IGCs use in COPD patients has been linked to an increased risk of pulmonary tuberculosis, not related to the use of oral steroids [53, 54]. Also, there are many less severe but more common side effects associated to the long-term use of IGS, such as thrush, hoarseness, bruising, hyperglycemia and modest effects on bone density [55, 56]. Additionally, the cost of IGCs is not low; in the US the daily use of fluticasone riches $194 a month [57].

This study suggests that not all COPD patients benefit from IGS, leading to a renewed interest in defining patients who could be managed with other therapies. It is possible that even patients with severe disease, when stable, can be managed for a period of time with bronchodilators alone [50].

## COPD in Non-Smokers

Of the patients with COPD, 25-45% have never smoked. In these patients, the most important identifiable risk factors include exposure to biomass fuel, occupational exposure to dust and fumes, history of pulmonary tuberculosis or chronic asthma, outdoor pollution and poor socioeconomic status. Approximately 3 billion people (half the world’s population) are exposed to smoke from biomass fuels, compared to 1.01 billion people who smoke tobacco; consequently, exposure to biomass smoke might even be the biggest risk factor for COPD globally, as it is in developing countries [58].

Biomass is a solid fuel used for heating and for cooking in open fire stoves, which is composed of plants (wood, coal, crop, twigs, and dried grass) and animal residues (dung). Developing countries burn about 2 billion kilograms of biomass per day [59, 60]. The smoke emitted contains a number of pollutants: particulate matter (PM_2.5_, PM_10_), carbon monoxide, nitrogen dioxide (NO_2_), sulfur dioxide, formaldehyde and polycyclic organic matter. These pollutants are similar to those present in tobacco smoke, and they start the inflammatory process in small airways and lung parenchyma [61]. In developing countries, about 50% of COPD deaths are related to biomass smoke, 75% of which are in women [62]. The World Health Organization declared the environmental pollution from biomass as one of the top 10 health risks, as it is responsible for 1.5 million deaths annually [63].

In Turkey, studies among non-smoking women exposed to biomass smoke have shown a direct relationship between the decline in FEV_1_ and exposure in hours/year to wood (province of Anatolia and Black Sea) and dung (East Anatolia) [64]. Over 80% of households in China, India, and Sub-Saharan Africa use biomass fuel for cooking, and in rural areas of Latin America the proportion varies between 30% and 75%. In developed nations like Canada, Australia and in western states of the US, the rising cost of energy has led to an increase in the number of households using wood and other biomass for heating [65].

Few studies have compared the phenotype of COPD in non-smokers to smokers. In a Mexican work, women who had COPD and had been exposed to smoke from biomass fuel had similar clinical characteristics, quality of life, and mortality to those with smoking-related COPD [66]. Shavelle and colleagues found that in the US, never-smokers had a reduction in life expectancy when compared to smokers [67]. Moran-Mendoza and colleagues reported that women with COPD due to biomass smoke have more pulmonary fibrosis, increased pigment deposition and greater pulmonary intimal thickening than women with COPD due to tobacco smoking [68].

Several questions need to be answered about this COPD phenotype, including the real burden in different countries, the clinical, radiological and functional characteristics, the cellular and immunological profiles, the prognosis and particularly, if the treatment should be the same. In this sense, there are no studies of the use of anticholinergics in these patients. Given that tobacco smoking has been considered the leading cause of COPD in developed countries, research protocols not related to tobacco are lacking.

## Tiotropium in Bronchial Asthma

According to various protocols, the short-acting muscarinic antagonist (SAMA), ipratropium bromide, can be used in multiple doses during asthma attacks [4, 69]. As a rescue drug, it is considered less effective than short-acting β_2_-agonists (SABAs), but when used together, ipratropium bromide produces a statistically significant improvement in lung function, and it is also an alternative for patients experiencing tachycardia, arrhythmias and tremor with the usage of SABA [70]. The role of tiotropium bromide (LAMA) has been less clear as a maintenance drug in bronchial asthma.

Even with the most recent therapeutic recommendations, up to 50% of asthmatic patients are not well controlled. The subgroup of patients require high doses of medications and still have persistent symptoms and constant exacerbations. There are many commonly used designations for this: refractory asthma, severe asthma, steroid-resistant asthma, steroid-dependent asthma, difficult-to-control asthma, poorly controlled asthma, fragile asthma and irreversible asthma [70].

The most recent term “severe refractory asthma”, comprises patients who remain difficult to control despite extensive re-evaluation and an appropriate observation period of at least 6 months by a specialist. Before confirming the diagnosis, however, phenotypic factors that contribute to refractory asthma should be recognized and properly treated [71]. Usually these patients are treated with LABAs and high doses of IGCs. GINA guidelines recommend combination therapy with other drugs, such as leukotriene modifiers, theophylline or monoclonal anti-IgE antibodies. Oral steroids are associated with severe side effects [72].

The non-neuronal cholinergic system is widely expressed in epithelial cells, eosinophils, submucosal gland cells, smooth muscle cells, and a variety of immune cells, including lymphocytes, macrophages and airway mast cells. This suggests that non-neuronal cholinergic signals may play an important role in the pathophysiology of bronchial asthma [73]. Therefore, the use of anticholinergic drugs seems promising in asthmatic patients not responding to IGCs.

In an exhaustive literature search of tiotropium in asthma, 149 papers published throughout 67 years were found, including just five randomized clinical trials and two open uncontrolled studies. The use of tiotropium in combination therapy with IGCs alone or IGCs plus LABAs in uncontrolled moderate to severe persistent asthma, showed improvement in lung function that was sustained even when IGCs were reduced and when LABA were discontinued. As a result, it could be considered beneficial to these patients, without adding safety concerns [74].

The TALC study showed that tiotropium (18 µg per day HandiHaler^®^) plus low-dose steroids was more effective than doubling the dose of steroids in improving morning and evening PEF (P ≤ 0.001) and in FEV_1_ (P ≤ 0.004). It was no less effective than salmeterol in improving lung function, and it had an acute bronchodilation effect in poorly controlled asthma [75]. In the work of Kerstjens and collaborators, using tiotropium (5 µg per day Respimat^®^) increased by 56 days the time to first severe exacerbation, with a 21% reduction in the risk of severe exacerbations versus placebo (P = 0.03) [76].

Although the improvement in lung function appears to be statistically significant, clinical studies have failed to show consistent improvement in respiratory symptoms, use of rescue medication or quality of life. This could be due to the size of the studies and perhaps to a ceiling effect [74]. A recent meta-analysis draws similar conclusions, but given its limitations, prospective double-blind, multicenter studies are required to validate the efficacy and safety of adding tiotropium to patients with uncontrolled asthma [77]. It should also be taken into account that more than 80% of patients with severe refractory asthma have poor adherence to controller medications, and the reasons that promote this behavior need to be solved before simply adding a drug [78].

The consistent improvement in lung function and the lack of significant side effects when adding tiotropium, probably led the Medicines and Healthcare Products Regulatory Agency (MHRA) in the United Kingdom, to the approval of Spiriva Respimat^®^ inhalation spray for asthma on September 13, 2014. In our country, Costa Rica, the Ministry of Health approved the Respimat^®^ inhaler for adult asthmatic patients with bronchial asthma who remain symptomatic despite the usage of IGCs in April 2015. GINA May 2015 guidelines consider tiotropium as an add-on therapy for steps 4 and 5, considering the benefits of improved lung function and increased time to severe exacerbation [79]. It is likely that the FDA will approve relatively soon the use of tiotropium for patients with severe persistent asthma uncontrolled with IGCs.

## Tiotropium in ACOS

Published reports indicate that there are a considerable number of patients over 50 years of age who have obstructive airway disease with features of a dual diagnosis of asthma and COPD [80]. Debate continues to whether or not COPD develops from asthma (Dutch hypothesis) or if both entities are completely independent (British hypothesis) [81, 82]. Recently, a joint effort between GOLD and GINA attempted to characterize an overlap syndrome (ACOS), which shares features of both diseases.

ACOS definition is purely descriptive; it refers to a subgroup of patients with persistent airflow limitation that concomitantly shows several features usually associated with asthma and several features usually associated with COPD. The fundamental point of identifying and properly treating these patients is that they seem to have a poorer prognosis. ACOS patients have frequent exacerbations, poor quality of life, fast decline in lung function and increased mortality, and they also consume more health resources than patients with asthma or COPD alone [83].

The basic treatment of ACOS is a combination of IGCs plus LABA and/or LAMA. Even if the overlap syndrome was acknowledged until recently, the possible benefit of tiotropium in patients with COPD and airway hyper-reactivity or concomitant asthma was pointed early [84, 85]. Magnussen and colleagues did a clinical 12-week, prospective, randomized double-blind, placebo-controlled, multicenter study that included 472 patients [86]. Patients receiving tiotropium achieve a statistically significant spirometric improvement in FEV_1_ compared to those in the placebo group (P ≤ 0.001, AUC_0 - 6 h_). There was also a significant reduction in the use of rescue medication in the tiotropium group. Tiotropium may be a useful bronchodilator in ACOS, added to IGCs with or without concomitant LABA, depending on the clinical and spirometric picture [86].

## Conclusions

Tiotropium bromide is a safe and efficient bronchodilator in the treatment of moderate to very severe COPD. It is plausible that this drug will have the same benefits in the treatment of COPD in non-smokers than in smokers, but this still requires demonstration. Tiotropium also has an expanding range of indications in bronchial asthma and ACOS. Given its well established anticholinergic long-acting mechanism, known therapeutic doses and the variety of delivery devices, tiotropium serves as a comparison for other bronchodilator molecules and as a reference for combination therapy. Tiotropium is to be expected as a protagonist in future trials of chronic obstructive lung conditions.
